# Flexible Teachers, Thriving Classrooms: A Unified Flexibility and Mindfulness (UFM) Model of Classroom Dynamics, Teaching Practices, and Teacher Burnout

**DOI:** 10.3390/bs16061018

**Published:** 2026-06-17

**Authors:** Katie Palmer, Ronald D. Rogge

**Affiliations:** 1 VigeoBx Consulting, Palm Desert, CA 92260, USA; katie@vigeobx.com; 2 Department of Psychology, University of Rochester, Rochester, NY 14627-0266, USA

**Keywords:** psychological flexibility, mindfulness, unified flexibility and mindfulness model, instructional strategies, behavior management, teacher burnout, acceptance and commitment therapy, relational density theory, student engagement, social and emotional competence

## Abstract

Within the conceptual framework of the Unified Flexibility & Mindfulness (UFM) model, the current study applied a contextual behavioral science lens to understanding the challenges and dynamics of classroom teaching in the United States. In particular, the study sought to highlight the specific flexibility processes linked to lower teacher burnout and to greater use of adaptive instructional and behavior management strategies—deepening the conceptualization and operationalization of teachers’ Social and Emotional Competence (SEC). Toward that end, a sample of 308 K-12 teachers (79% female, 85% white, M_age_ = 42 years old) with an average of 13 years of teaching experience completed a relational task (RT) indirectly assessing relational thinking about students along with teacher-report measures of: (1) their own use of 14 forms of mindful flexibility (and distracted, reactive inflexibility) in the classroom, (2) their conscious perceptions of student engagement and disaffection with learning, (3) their use of adaptive instructional and behavior management strategies, and (4) a measure of work-related and student-related burnout. Exploratory network analyses largely supported the Unified Flexibility and Mindfulness model shaping teachers’ functioning in the classroom. The results further highlighted unique links from categorical thinking on the RT (i.e., viewing all positive or negative adjectives as essentially the same in students) to greater burnout and unique links from more nuanced thinking on the RT (i.e., the ability to see negative and positive traits coexisting in students) to greater perceptions of both student engagement and disaffection. Teachers’ engagement of committed action and self-as-context (maintaining a broader perspective in the face of disruptive behavior) along with perceptions of greater student engagement emerged as some of the most robust predictors of using adaptive classroom strategies. In contrast, teachers’ engagement in fusion and inaction (along with perceptions of greater student disaffection and lower student engagement) emerged as the most robust predictors of teacher burnout. Implications are discussed.

## 1. Introduction

### 1.1. Background and Literature Review

#### 1.1.1. The Teacher Burnout Crisis

Teaching children and adolescents is critical work, but it can also be extremely challenging and difficult. Even motivated and hard-working students have off days, and so, in addition to providing engaging and dynamic instruction, teachers are bombarded with difficult and challenging behavior from their students on a daily basis. Given this, it is less surprising that teachers are struggling with unhealthy levels of stress, burnout, and attrition (e.g., Clunies-Ross et al., 2008; Harmsen et al., 2018). For example, in a 2012 study of 236 teachers and staff, 75% of the sample met clinical levels of anxiety, depression, or stress (Jeffcoat & Hayes, 2012). The constant barrage of demands and the corresponding levels of stress, compassion fatigue, and burnout that teachers experience (e.g., Mrstik et al., 2019; Schlichte et al., 2005; von der Embse et al., 2019) can represent a significant drain on the teachers’ own internal resources, potentially tainting their perceptions of students and even making it more challenging to engage effective instructional and behavioral management strategies. Consistent with these findings, the US education system is experiencing a crisis of teacher shortages, with estimates of over 411,000 teaching positions in the US currently unfilled or filled by uncertified teachers (Tan et al., 2025).

Underlying this crisis, an expansive body of research has found that student misbehavior affects teacher stress, well-being, confidence, and has a negative impact on student learning and academic achievements (Friedman, 1995; Hastings & Bham, 2003; Herman et al., 2018; Kyriacou, 2001; Tobin & Sugai, 1999; Skaalvik & Skaalvik, 2010; Tsouloupas et al., 2010; Sutherland et al., 2008). For example, Clunies-Ross et al. (2008) found that even minor student misbehavior such as talking out of turn affects teacher levels of stress (increasing the stress response) as well as their well-being and confidence (decreasing these values). Additional research supports relationships between teacher stress, teacher attrition, and teachers’ negative perceptions of students (Harmsen et al., 2018; von der Embse et al., 2019). From a contextual behavioral science (CBS) perspective, disruptive student behavior represents difficult and challenging experiences for teachers to navigate. Thus, their engagement of mindfully flexible (or distracted, reactive, inflexible) behavioral repertoires in response could serve to buffer (or exacerbate) the impact of challenging student behavior on the classroom dynamics.

#### 1.1.2. Perceptions of Student Engagement/Disaffection

A large body of previous work has indicated that student engagement is reciprocally linked to classroom dynamics including teachers’ behavior management and teaching strategies as well as their own experiences of burnout (see Fredricks et al., 2004 for a review). For example, teachers’ perceptions of low student engagement and high student disaffection (along with perceptions of disruptive behavior) have been robustly linked to greater stress and burnout (Jennings & Greenberg, 2009; Skaalvik & Skaalvik, 2010). Lower student engagement and greater student disaffection have also been linked to teachers using more controlling or reactive responses to disruptive behavior (Reeve, 2012; Skinner & Belmont, 1993; Skinner et al., 2009). Teachers’ perceptions of student engagement/disaffection have also been linked to their instructional practices, suggesting that teachers tend to adapt their teaching strategies in response to students’ levels of engagement (Pianta et al., 2012; Van den Berghe et al., 2016). Taken together, these findings highlight that teachers’ perceptions of student engagement and disaffection might serve as critical lenses through which classroom dynamics, instructional practices, and teacher well-being are shaped.

#### 1.1.3. Addressing the Crisis with Social and Emotional Competency (SEC)

The current teacher burnout crisis has drawn attention to the critical need to support and develop the Social and Emotional Competency (SEC) of teachers (Jennings & Greenberg, 2009) to not only promote their own well-being but to also create prosocial classrooms in which students can thrive. To promote the SEC among educators, a number of previous studies have provided ACT-based interventions to teachers (e.g., Biglan et al., 2013; Gillard et al., 2021; Paliliunas et al., 2023). These studies demonstrated declines in stress and burnout as well as improvements in self-efficacy, self-compassion, well-being, and specific dimensions of psychological flexibility (e.g., drops in experiential avoidance, increases in mindful awareness and valued living; Biglan et al., 2013). Toward the same end, previous studies have delivered mindfulness-based interventions to teachers (e.g., Flook et al., 2013; Jennings et al., 2017; Roeser et al., 2013; see Emerson et al., 2017 for a review), demonstrating improvements on classroom organization, adaptive emotion regulation, self-compassion, psychological distress, burnout, and mindfulness. These ACT and mindfulness treatment studies also uncovered correlational links between a handful of more specific mindful flexibility dimensions and the various outcomes examined. Thus, these approaches highlight the importance of cultivating awareness, acceptance, and value-guided behavior among teachers. However, despite this promising intervention literature, relatively little work has examined the broad compliment of behavioral processes that could serve as potential treatment mechanisms to explain the benefits of ACT and mindfulness-based interventions on the day-to-day functioning of teachers in the classroom.

#### 1.1.4. Enhancing Understanding of SEC with the UFM Conceptual Framework

Drawing from the mindfulness and contextual behavioral science (CBS) literature, the Unified Flexibility and Mindfulness model (UFM; Rogge & Daks, 2021) offers a promising multifaceted conceptual framework for understanding and promoting greater SEC within teachers—operationalizing that broader construct into 14 distinct processes. Those 14 distinct behavioral repertoires have been shown to play critical roles in helping individuals navigate difficult and challenging thoughts, feelings, and experiences in their daily lives across a series of experimental studies (Levin et al., 2012), across hundreds of correlational studies (Daks & Rogge, 2020), and have emerged as potential treatment mechanisms across dozens of treatment studies (e.g., Macri & Rogge, 2024). The UFM also offers an optimized tool to assess and track those processes (the UFM scale; Rogge & Rasmussen, 2024). Most notably, the UFM framework is grounded in a broad range of highly effective clinical interventions and in-session techniques, exercises, metaphors, and visualizations that have been developed to target those processes within the mindfulness-based intervention (e.g., Mindfulness-Based Stress Reduction; MBSR; see Grossman et al., 2004) and the Acceptance and Commitment Therapy (ACT; Hayes et al., 1999, 2011) literatures. Thus, the UFM framework could fill a critical need in the work on understanding and promoting teachers’ SEC by offering a wealth of well-validated and rigorously tested intervention techniques directly linked to the UFM processes. The UFM has received: (1) cross-sectional support in diverse samples using both exploratory and confirmatory analyses (Rogge & Daks, 2021; Parker et al., 2024), (2) cross-cultural support across US, Japanese, Taiwanese, and Chinese samples (Rogge et al., 2024), and (3) preliminary longitudinal support in 2-wave correlated change analyses (Rogge et al., 2026). Thus, the current study sought to apply a CBS lens to the task of understanding and promoting greater teacher SEC by applying the UFM framework to this emerging area of research.

#### 1.1.5. Enhancing Understanding of SEC via Relational Density Theory (RDT)

To further scaffold this work within a CBS framework, the current study sought to expand beyond self-report data by using a relational task based on the RDT literature (e.g., Belisle & Dixon, 2020) that indirectly assesses teachers’ relational thinking about students. RDT proposes that the higher the mass (or density and volume) of a given network of associated words/meaning, the more resistant to change those related meanings are (Belisle & Clayton, 2021; Belisle & Dixon, 2020). For instance, a teacher associating loud, boisterous, distracted students tightly with unsuccessful or defiant students would be suggestive of categorical relational thinking. This is because a highly dense network of student negative traits in a teacher’s perceptions (along with a separate highly dense network of positive traits) could lead teachers to view students in a oversimplified manner—seeing them as globally good or globally bad rather than viewing them in a more nuanced manner that could allow the perception of unique combinations of strengths and weaknesses in each student.

To assess the density and volume of a network of relations, researchers have used relational tasks in which respondents were asked to rate the level of similarity of all possible pairs within sets of positive and negative adjectives concerning some target group (e.g., Hutchison et al., 2023). Average ratings of each pair could then be used to create a proximity/distance matrix across the various adjectives and the density of that matrix (at a group level) could be visualized with multidimensional scaling procedures. Thus, these studies have largely been conducted: (1) within controlled, laboratory-based paradigms, (2) typically relying on relatively small samples, and (3) have focused on evaluating the density and volume of network relations exclusively at a group level, often contrasting results in treatment versus control groups or pre-treatment versus post-treatment network relations (e.g., see Belisle & Dixon, 2020; Hutchison et al., 2023). Although this innovative work has laid a solid foundation, its focus on group-level results has precluded its application to understanding individual differences in relational thinking and how those differences may relate to meaningful psychological and behavioral outcomes. The current study sought to extend work on RDT and apply it to the teacher burnout crisis by having teachers complete a student-focused relational task. To bring the focus of RDT down to the individual level, the study built on recent pilot work (Palmer, 2025) developing a data analytic strategy for relational task data that can assess the relational thinking styles of individual teachers.

### 1.2. The Current Study

The current study sought to enrich and deepen our understanding of teachers’ SEC and the behavioral processes that could potentially foster resilience and prevent burnout as teachers manage the day-to-day chaos and stress of classrooms. More specifically, the study examined a range of classroom dynamics within the broader conceptual frameworks of the UFM and RDT models, thereby drawing upon over 20 years of psychology research and clinical resources that could be brought to bear in response to the current teacher burnout crisis. Toward that end, an online sample of 308 teachers completed measures of their (1) levels of burnout, (2) teaching and behavior management strategies, (3) self-reported perceptions of students’ engagement and disaffection, (4) relational thinking about students (indirectly assessed with a relational task), and (5) a comprehensive measure of the degree to which they engage the 14 behavioral processes within the UFM model in the context of their classrooms. Exploratory psychological network analyses were used to uncover the unique links between the components of the UFM model and the classroom dynamics examined.

The current study also extended the emerging literature on RDT by recruiting a large online sample that could support the application of Exploratory Factor Analysis (EFA) techniques to data from a relational task. This allowed us to develop individual-level indices of teachers’ relational thinking using a set of student-focused adjectives. Thus, this approach enabled, for the first time, the assessment of teachers’ relational thinking about students at an individual level (rather than a global group level) and the examination of how those relational thinking styles might demonstrate correlational links to their engagement in psychologically flexible versus inflexible behavioral repertoires, as well as to their instructional practices, perceptions of students, and experiences of burnout. Notably, the indirect nature of the relational tasks underlying this approach also offered a source of information on classroom dynamics beyond direct self-report data from teachers.

### 1.3. Hypothesis Building

Given the marked novelty of examining teachers’ use of mindful/flexible and distracted/rigid/inflexible behavioral repertoires in their classrooms as critical processes that could shape classroom dynamics, we chose to take a stepwise approach to examining those links. Thus, we examined two separate network models.

#### 1.3.1. Model 1—Testing Omnibus Links of Global In/Flexibility

**Figure 1:**
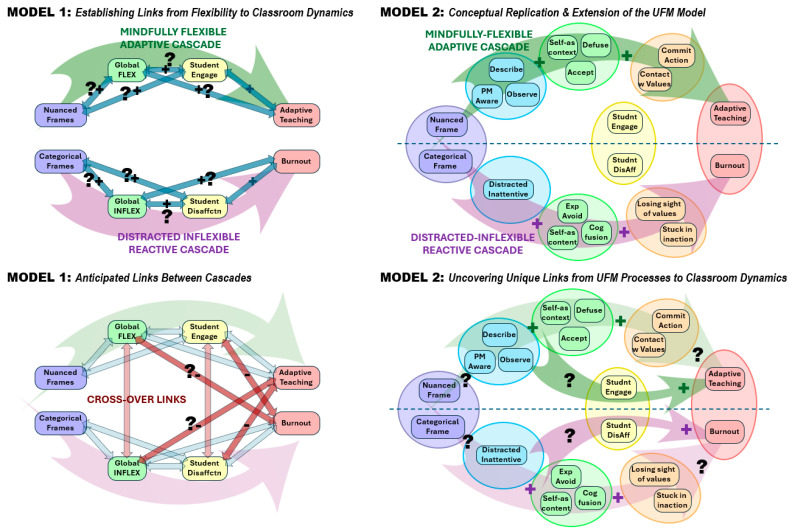
Anticipated Results from the Exploratory Network Analyses. NOTE: The thinner blue and red, double-headed arrows represent the anticipated positive and negative unique links (i.e., edge-weights) to emerge in the planned network analyses. The thicker, green and purple, curved arrows highlight broader cascades of positive links across multiple constructs that are anticipated to emerge in the analyses. The question marks highlight the novel questions to be addressed in the analyses. The “+” and “-” signs indicate expected positive and negative links respectively. The dashed lines in model 2 are there to help distinguish the adaptive and reactive cascades.

In our first model (Figure 1), we explored teachers’ global engagement of mindfully-flexible behavioral repertoires and their global engagement of distracted-inflexible repertoires in the classroom using composite scores. This focused the model on examining the unique links to the shared variance among the 8 dimensions of mindful-flexibility as well as the shared variance among 6 dimensions of distracted-inflexibility. Thus, Model 1 highlighted how mindfully-flexible behavioral repertoires, as a set, and distracted-inflexible behavioral repertoires were broadly linked to the classroom dynamics examined (indicated with the “?” characters in Figure 1). From this perspective, Model 1 functioned as an initial omnibus test of whether teachers’ engagement of mindful-flexible and distracted-reactive-inflexible behavioral repertoires in the classroom (in response to the normative chaos of classroom teaching) might shape classroom dynamics. As seen on the left side of Figure 1, we anticipated that teachers maintaining more nuanced relational thinking toward students might promote resilience in the classroom by helping teachers (1) engage in more in psychologically flexible and mindful behavioral repertoires in response to classroom disruptions, (2) linking to greater perceptions of student engagement, (3) supporting greater value-driven behavior management and teaching even in the face of challenging and obstructive behavior, and (4) promoting lower levels of burnout. In contrast, we anticipated that greater entrenchment in categorical relational thinking toward students would be linked to (1) greater engagement of defensive-reactive-inflexible behavioral repertoires in response to classroom disruptions, (2) greater perceptions of student disaffection, (3) linking to more reactive behavior management and teaching strategies, and (4) greater burnout. We also anticipated unique links from global engagement of flexibility and inflexibility skills to higher perceptions of student engagement and disaffection (respectively), and to the outcomes of adaptive teaching skills and burnout.

#### 1.3.2. Model 2—Exploring Links to Specific UFM Processes

As shown on the right side of Figure 1, we built a second model to compliment the findings of the first model by including all 14 distinct dimensions of mindful-flexibility within the UFM framework. This effectively shifted the shared variance of mindful-flexibility and distracted-inflexibility processes (examined in the first model) into the unique edge weights emerging among the 14 UFM processes (forming the UFM framework). Thus, Model 2 served to not only replicate and extend findings supporting the UFM framework, but also to uncover the unique links from the 14 UFM processes to the classroom dynamics variables examined.

#### 1.3.3. The UFM Mindfully-Flexible Adaptive Cascade

**Figure 2:**
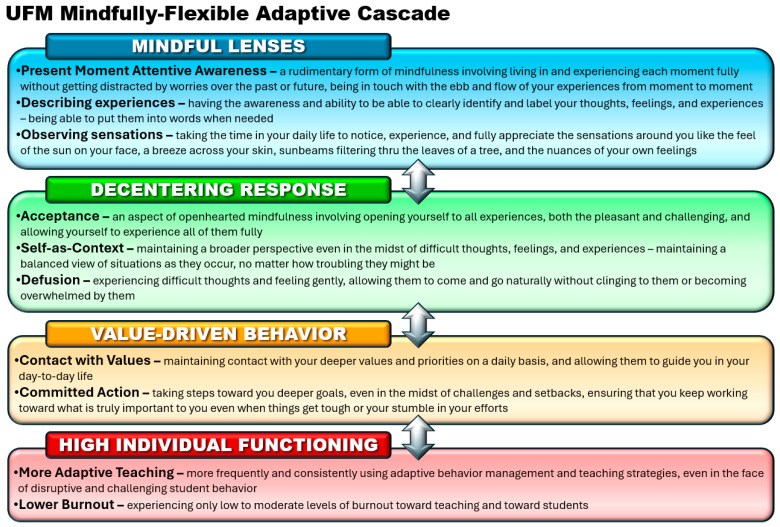
The Mindfully-Flexible Adaptive Cascade Proposed by the UFM Model.

As shown in Figure 2, the UFM model posits that a set of processes function as mindful lenses that allow teachers to detect potentially difficult and challenging experiences (e.g., disruptive or challenging student behavior) as they arise. Thus, engaging those repertoires would promote (i.e., show strong proximal links to) greater mindful decentering from those experiences (rather than defensively reacting to them), allowing teachers to engage a more accepting stance, maintain a broader perspective, and gently experience those experiences without clinging to them—even in the midst of student disruptions. The model then posits that greater decentering from difficult and challenging experiences would, in turn, promote greater value-driven behavior even in the midst of classroom chaos. Thus, teachers would be able to maintain contact with their deeper values and take committed action toward their deeper goals even in the face of setbacks and disruptions. Finally, the model posits that greater levels of value-driven behavior would promote more effective individual functioning (i.e., engaging in more effective teaching and behavior management strategies and experiencing lower feelings of burnout).

#### 1.3.4. The UFM Distracted-Inflexible Reactive Cascade

**Figure 3:**
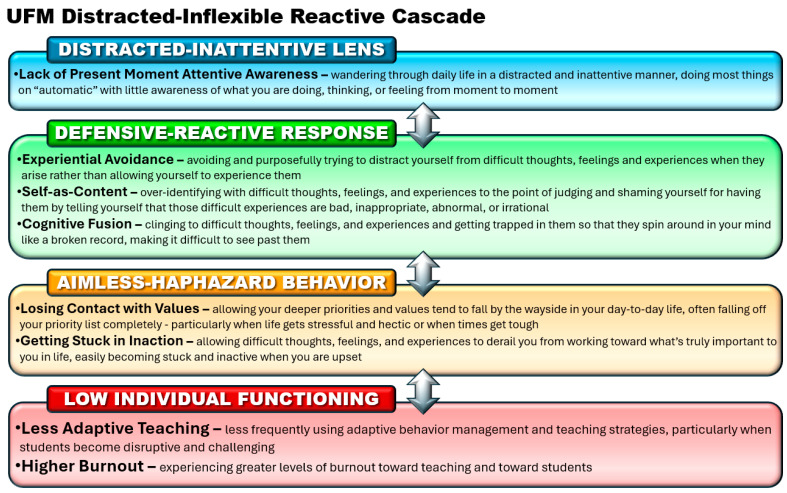
The Distracted-Inflexible Reactive Cascade Proposed by the UFM Model.

As seen in Figure 3, the UFM model also posits a disruptive cascade in which high engagement in distracted inattention to the present moment increases the likelihood of defensively reacting to difficult or challenging student behavior (i.e., avoiding those situations, judging and shaming oneself for them, and getting trapped in thoughts and feelings about them). The UFM model further posits that those defensive reactions would be robustly linked to aimless and haphazard behavior in the face of problematic student behavior (i.e., routinely losing contact with deeper values in the stress and strain of day-to-day classroom dynamics and getting stuck in inaction in the face of classroom chaos). Finally, frequent aimless and haphazard behavior would be linked to poorer individual functioning (less frequent/consistent use of adaptive teaching strategies and higher burnout).

#### 1.3.5. The Transactional Nature of the UFM Links

The arrows presented in Figure 2 and Figure 3 are all double-headed as the UFM model posits that these associations are likely to be bidirectional. The model also acknowledges that while the behavioral repertoires in one stage of the model will show some of their strongest links to one another and to their adjacent stages, they are likely to show links to subsequent stages as well, given the interconnected nature of these repertoires. Consistent with these points, despite the stepwise nature of the UFM model, the model does not assert that the path to developing mindful flexibility in one’s life (or even flexibly responding to a specific difficult event) is a perfectly linear path, recognizing instead that individuals likely shift back and forth between various stages.

As shown in the lower right panel of Figure 1, we anticipated that unique links would emerge between individual dimensions of mindful flexibility and the various aspects of classroom dynamics assessed (indicated by the question marks in that figure). However, as this was one of the first studies to apply the UFM model to classroom dynamics, these analyses were exploratory. We anticipated that teachers engaging adaptive mindfully-flexible processes would be linked to better classroom dynamics and lower burnout whereas teachers engaging distracted-inflexible processes would be linked to poorer classroom dynamics and higher burnout. We also anticipated that teachers’ self-reported perceptions of student engagement and disaffection would likely show strong unique links to our outcomes of adaptive teaching and burnout, potentially functioning as an intermediate mechanism between the UFM processes and those outcomes. Despite those broader hypotheses, we could not know in advance which of the UFM processes would emerge as the most critical in a classroom environment or show robust links to specific classroom dynamics.

## 2. Method

### 2.1. Participants

A total of 308 K through 12 teachers completed the survey. Respondents were an average of 42 years old (SD = 11.2) and identified primarily as female (79%), with 19% male, and 2% nonbinary, fluid, or agender and white (85%), with 6% black, 5% Hispanic/Latinx, 1% biracial, 1% Asian, and 1% other. A majority (88%) reported having bachelor’s or masters degrees and 59% of the sample reported annual household incomes of below $100,000.

### 2.2. Procedure

The study involved a 25–30 min online survey. The first webpage of the survey provided an information sheet to obtain informed consent. The second webpage then collected eligibility information, sending ineligible respondents to an end-of-survey thank you page so as to avoid collecting substantive data from those ineligible respondents. Respondents were eligible to participate if they were currently teaching kindergarten through 12th grade students, were fluent in English, and were between the ages of 18 and 70. Individualized feedback was provided at the end of the survey as the primary recruitment incentive.

#### 2.2.1. Recruitment

The recruitment materials provided the study title, “The Understanding Teachers’ Perspectives Survey”, along with some basic information about the study (e.g., entirely voluntary, completed online, involving a 25–30 min survey) and a link to the online survey. Participants were recruited from a range of sources including: the Prolific crowdsourcing platform (70%), the ResearchMatch participant registry (25%), local school districts (4%), and other sources with lower yields (1%). Individualized feedback based on survey responses was provided at the end of the survey as the primary recruitment incentive. Participants recruited through Prolific were also given a $2.60 recruitment incentive.

#### 2.2.2. Relational Task

Using an approach developed to assess the density of relational networks (i.e., perceptions; Belisle & Clayton, 2021, Paliliunas et al., 2023), teachers rated 66 unique pairwise combinations of 12 adjectives (angry, defiant, disrespectful, distracted, loud, unsuccessful, thoughtful, cooperative, respectful, focused, quiet, successful) on a 10-point response scale (not at all similar, a tiny bit similar, a little similar, somewhat similar, moderately similar, fairly similar, quite similar, very similar, extremely similar, completely the same) using the stem, “STUDENT TRAITS: In your experience, how closely related are these two student characteristics?” The adjectives were developed in a focus group of behavior analysts who had experience developing other relational tasks as a part of their graduate training. The adjectives were specifically selected to represent a balanced set of opposing positive and negative student traits (as opposed to behaviors) centered around the concepts of successful and unsuccessful students. Responses on a set of 34 internally consistent pairs in which positive adjectives were paired with negative adjectives were averaged (α = 0.970) such that higher scores reflect greater nuanced thinking (i.e., seeing negative student traits as being able to coexist and share similarity with positive student traits). Consistent with the high internal consistency obtained for this subscale, an Exploratory Factor Analysis (EFA; using principle axis factoring extraction with direct Oblimin rotation) on those 34 item-pairs suggested a dominant first factor accounting for 51.6% of the variance. Specifically, the EFA yielded a first eigenvalue (ev1 = 17.557) over eight times greater than the second eigenvalue (ev2 = 2.105) which was part of the remaining linear skew (ev3–6: 1.247, 1.072, 0.848, 0.842). As seen in Supplemental Online Table S1, these 34 item pairs all demonstrated appropriately strong factor loadings on that dominant factor. Similarly, responses on a set of 29 internally consistent pairs linking positive adjectives with other positive adjectives and negative adjectives with other negative adjectives were averaged (α = 0.949) such that higher scores reflect greater categorical thinking (i.e., seeing all negative adjectives as essentially the same and all positive adjectives as essentially the same—a pattern of responding suggestive of black and white, categorical thinking). A subsequent EFA on the items of this subscale also suggested a dominant first factor (ev1–6: 12.199, 2.723, 1.443, 1.065, 0.883, 0.824). As seen in Supplemental Online Table S2, these 29 item pairs all demonstrated appropriately strong factor loadings on that dominant factor. This novel method of scoring responses on an RT at an individual level was recently successfully piloted as a method of representing relational frames from RT data in a manner enabling correlational analyses (Palmer, 2025).

### 2.3. Measures

Unless otherwise indicated, the self-report scales instructed teachers to focus on the last 2 weeks and were presented with common 6-point response sets (e.g., not at all, a little, somewhat, quite a bit, very much, extremely). Scales and subscales were scored by averaging responses across items (thereby yielding scores ranging from 1 to 6) such that higher scores reflect higher levels of the construct assessed by each scale. All Cronbach alpha coefficients presented were estimated within the current sample.

#### 2.3.1. Mindful Flexibility in the Classroom

**Figure 4:**
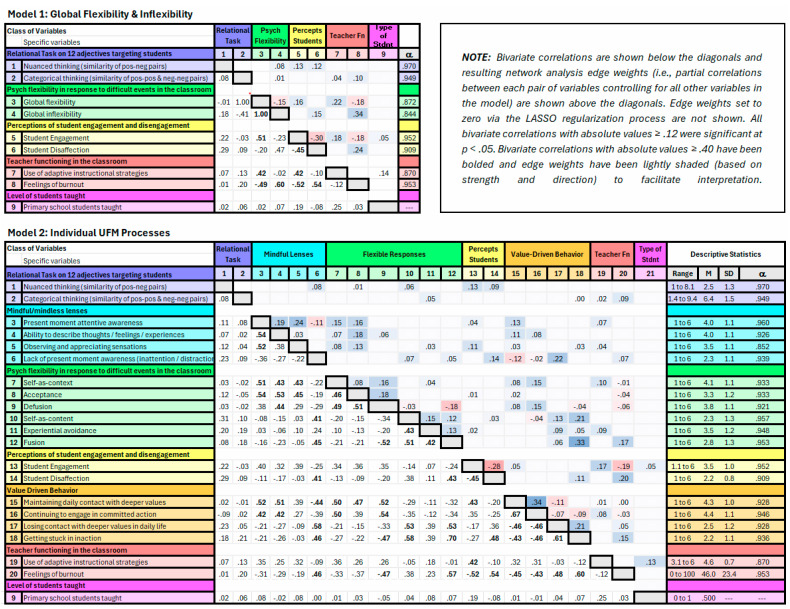
Bivariate Correlations, Network Analysis Edge Weights, Means, Standard Deviations, and Cronbach Alpha Internal Consistency Coefficients for All Variables across Both Models.

To assess teachers’ engagement of flexible and inflexible behavioral repertoires as they instruct and interact with students in their classrooms, teachers completed the Unified Flexibility and Mindfulness (UFM) measure (Rogge & Daks, 2021). This scale contains the 12 (5-item) subscales of the Multidimensional Psychological Flexibility Inventory (MPFI; Rolffs et al., 2018) along with 2 additional IRT optimized subscales conceptually drawn from the mindfulness literature. To focus the measure on their engagement of mindful flexibility (and rigid, mindless inflexibility) behavioral repertoires in the classroom, the original and unaltered UFM items were presented with the stem, “IN THE LAST 2 WEEKS, when I was in my classroom with students…” As shown in Supplemental Online Table S3, the UFM scale focused on how teachers’ behavior in the classroom continued to support a 14-factor structure in a CFA that gave adequate fit (χ^2^(2254) = 3781, *p* < 0.001; CFI = 0.929; SRMR = 0.049, RMSEA = 0.047). Consistent with these findings, the UFM subscales demonstrated robust internal consistency in the current sample with Cronbach alpha coefficients ranging from 0.854 to 0.959 (M_α_ = 0.933; see Figure 4). Following validated supplemental methods of the scoring of the UFM scale (Rogge & Daks, 2021), the positive subscales were then averaged to create a global index of mindful flexibility (*α* = 0.872; M = 3.92; SD = 0.80) and the negative subscales were averaged to create a global index of inflexibility (*α* = 0.844; M = 2.60; SD = 0.90).

#### 2.3.2. Student Engagement and Disaffection

Teachers completed the Engagement Versus Disaffection with Learning scale (EvsD; Ritoša et al., 2020). Responses on the 10 behavioral and emotional engagement items were averaged to create a student engagement score (*α* = 0.898) such that higher scores reflect higher student engagement. Similarly, responses on 12 internally consistent behavioral and emotional disaffection items were averaged (*α* = 0.909) to create a student disaffection score such that higher scores reflect higher student disaffection.

#### 2.3.3. Teaching Strategies

Participants completed 41 items of the Classroom Strategies Scale—Teacher form (CSS-T; Reddy et al., 2015) which assesses 3 instructional strategies (performance feedback *α* = 0.859, promoting thinking *α* = 0.841, instructional delivery/student focus *α* = 0.834) and 4 behavioral management strategies (praise *α* = 0.879, corrective feedback *α* = 0.807, prevention management *α* = 0.749, directives/transitions *α* = 0.855). Given the robust correlations among the CSS-T subscales, we created an overall teaching strategy composite by averaging across all subscales (*α* = 0.870).

#### 2.3.4. Burnout

Teachers completed 12 items from 2 subscales of the abbreviated Copenhagen Burnout Inventory (CBI; Barton et al., 2022). The original CBI client-related burnout subscale was developed for human-service professions working with patients or clients. However, multiple studies have validated teacher-focused versions of the Copenhagen Burnout Inventory using student-related burnout items (e.g., Fiorilli et al., 2015; Milfont et al., 2008; Piperac et al., 2021). Aligning with this previous work, items of the CBI patient subscale were slightly modified to focus on students by simply replacing the word “patients” in each item with “students”. Thus, the CBI assessed work-related burnout (*α* = 0.919) and student-related burnout (0.935). As shown in Supplemental Online Table S4, the two CBI subscales used in the current study continued to support a two-factor structure in a Confirmatory Factor Analysis (CFA) that gave excellent fit (χ^2^(50) = 146, *p* < 0.001; CFI = 0.972; SRMR = 0.034, RMSEA = 0.079). As those two subscales demonstrated marked collinearity in their subscale scores (*r* = 0.80) and in the CFA (*r* = 0.84), we created a composite score assessing global classroom burnout by averaging across all of the items (*α* = 0.953). Averages on that composite were converted to the common 0 to 100 range used for CBI scores (with higher scores reflecting greater burnout). Although clinical bands of severity for the CBI have yet to be empirically established (see Hansen & Virden, 2022), the field has accepted and widely adopted the convention that scores from 50–74 represent moderate burnout, scores from 75–99 represent high burnout, and maximum scores of 100 represent severe burnout (e.g., Creedy et al., 2017; Hansen & Virden, 2022). The current study made use of those severity bands to help describe levels of burnout in the sample, but treated burnout as a continuous variable in the main network analyses.

### 2.4. Analytic Strategy

IBM SPSS Statistics for Windows, Version 29.0 (IBM Corp., Armonk, NY, USA) was used to prepare the raw data from the Qualtrics survey platform for use in R (within RStudio, Version 2024.04.2+764; Posit PBC, Boston, MA, USA) and to generate bivariate correlations and sample descriptive statistics.

#### 2.4.1. Data Exclusion

Ineligible subjects were screened at the start of the baseline survey immediately after informed consent and were actively prevented from completing any more of the study. Between 14 March 2025 and 5 June 2025, a total of 471 respondents started the online survey of this project. Of those respondents, a total of 315 completed at least 70% of the survey (i.e., completing at least 238 of the roughly 340 items) for a survey completion rate of 67%. Among the 315 completers, 2 respondents completed the survey at an excessively fast rate (26+ questions per minute—more than three times the average rate) and were therefore excluded from the final data for rushing. Of the remaining 313 respondents, 5 made mistakes on 6 or more of the 10 directed questions that were distributed throughout the survey (i.e., “To show you are paying attention, please leave this question unanswered”) and were therefore excluded for lack of sufficient attention. This data exclusion process therefore resulted in the final sample of 308 respondents whose data will be used in the analyses.

#### 2.4.2. Missing Data

The final sample of 308 respondents had only nominal levels of missing data (0.9%) on the variables used in the network analyses. In addition, Little’s MCAR test suggested that the pattern of missing data did not significantly differ from the pattern expected from Missing Completely At Random (MCAR; χ^2^(1777) = 1297, *p* = 0.999). Taken together, these findings markedly attenuate the risk of that missingness influencing the results.

#### 2.4.3. Multivariate Normality

As the findings of network analyses can be influenced by a lack of multivariate normality (see Epskamp & Fried, 2018), we evaluated the multivariate normality of our data with the MVN package in R (Korkmaz et al., 2014) prior to conducting our network analyses. Those analyses identified significant multivariate skew and kurtosis (a common issue with self-reported survey data). Following current best practices (Epskamp & Fried, 2018) we used the huge package in R (Zhao et al., 2012) to conduct a nonparanormal transformation (Liu et al., 2009).

#### 2.4.4. Network Analyses

The network analyses were conducted within R (version 4.0.3) using the estimateNetwork function in the bootnet package (Epskamp & Fried, 2021; version 1.9) to extract the model and the predict function in the MGM package (Haslbeck & Waldorp, 2015) to generate multiple *R*^2^ estimates for each node. Following current best practices (Epskamp & Fried, 2018), we estimated a gaussian graphic model on Spearman’s rank-correlations to adjust for the ordinal nature of Likert-response data. To minimize the risk of interpreting spurious or unstable edge-weights (Brunner & Austin, 2009; Babyak, 2004), we used regularization with the least absolute shrinkage and selection operator (LASSO; Tibshirani, 1996) to help simplify and focus the network analyses on more stable and parsimonious solutions. This form of regularization applies increasing penalties (i.e., shrinking edge weights) to models with larger numbers of parameters and sets notably small edge weights to zero, thereby helping to minimize the interpretation of spurious effects. Thus, LASSO regularization was utilized with the Extended Bayesian Information Criterion (EBIC; Chen & Chen, 2008) selection method (Epskamp & Fried, 2018). We also set the tuning parameter at a conservative 0.5 to favor more parsimonious (i.e., sparse) models. The qgraph package (Epskamp et al., 2012; version 1.9) was used to create graphs of the network results. The current study only focused on the centrality estimates of “strength” and “expected influence” as those can be more reliably estimated and have clearer interpretations when network analysis is applied to variables rather than social networks (see Bringmann et al., 2019; Hallquist et al., 2021).

#### 2.4.5. Accuracy and Stability of Network Estimates

Using the netSimulator function within the bootnet package for R (Epskamp & Fried, 2021), we compared the results obtained within our data to results from 1000 simulations across a number of sample sizes. This allowed us to determine the relative power and stability of the network results. We also used the bootnet function of the bootnet package to generate 1000 nonparametric bootstrapped samples to obtain 95% confidence intervals for the edge-weights estimated in our models. Finally, we used the bootnet function to generate 1000 case-dropping bootstrapped samples, thereby allowing us to investigate the stability of the centrality estimates from our network models.

### 2.5. Transparency and Openness

All study materials and procedures were evaluated and approved as a minimal risk study by the University of Rochester Research Subjects Review Board and are available on the osf.io listing for this project (citation and link removed) along with (a) a preregistration for this article, (b) our R network analysis code, (c) our SPSS syntax, and (d) our final R and SPSS data sets (available upon reasonable request). In the preregistration we report how we determined our sample size, all data exclusions, all manipulations, and all measures in the study.

## 3. Results

### 3.1. Sample Descriptives

The 308 teachers surveyed ranged from 1 to 36 years of teaching experience (M = 13.6 years, SD = 8.8) and primarily taught fulltime (91%) at public schools (81%), with 8% at secular private schools, 8% at non-secular private schools, and 3% at other types of schools. Roughly 41% of teachers reported teaching a single grade level as a majority (59%) taught multiple grade levels (M = 2.9 grade levels, SD = 2.3 grade levels). Approximately 50% of teachers reported teaching at least one elementary school grade level (i.e., kindergarten to 5th grade), 28% teaching at least one junior high grade level (i.e., 6th to 8th grade), 42% teaching at least one high school grade level (i.e., 9th to 12th grade), and 26% reported currently teaching special education (those percentages total to more than 100% as those are overlapping groups). As shown by the means presented in Figure 4, the teachers in the sample reported notable levels of burnout toward work and students, with 93 teachers (31%) reporting moderate burnout (i.e., CBI scores from 50–74 out of 100), 38 teachers (13%) reporting high burnout (i.e., CBI scores from 75–99), and 2 teachers (0.7%) reporting severe burnout (i.e., maximum CBI scores of 100). Taken as a set, these findings suggest that our recruitment efforts were effective in curating a diverse sample of teachers representing a wide range of teaching settings and experiences.

### 3.2. Network Findings

#### 3.2.1. Occupational Controls

Given the wide range of schools, teachers, and grade levels included in the current study, we ran preliminary versions of Models 1 and 2 that included dichotomous variables coding school type (public school = 1, all others = 0), grade levels taught (primary school students = 1, no primary school students = 0), and special education teaching (teaching special education = 1, not teaching special education = 0). The variable coding primary school teaching was the only control variable to demonstrate unique links to the other variables in the models containing these controls. Thus, to control for these occupational variables while minimizing unnecessary collinearity in the models, we retained the primary school teaching in the models presented.

#### 3.2.2. Accuracy and Stability Checks

As shown in Supplemental Online Figure S1A,B, the results obtained from simulated datasets demonstrated reasonable levels of sensitivity and specificity for detecting meaningful edge weights and discriminating them from spurious edge weights (both above 0.75 on average for samples of 308 subjects). In addition, the results from those simulated datasets demonstrated robust correlations with the edge weight estimates from the actual data (correlations above 0.85 on average for samples of 308 subjects), suggesting reasonably high levels of stability for the current findings. Extending this, the centrality estimates of expected influence and strength (see Supplemental Online Figure S1C,D) also demonstrated robust correlations with the corresponding centrality estimates from the actual data (correlations above 0.80 for samples of 308 subjects), suggesting similar stability for those centrality findings for each model. As shown in Supplemental Online Figure S2A,B, confidence intervals estimated from non-parametric bootstrapping in the current sample helped to clarify the relative precision of the edge weight estimates. As shown in Supplemental Online Figure S2C,D, the case-dropping bootstrapping results further suggested that even dropping the bootstrapped sample size down to 70% of the current sample continued to yield centrality estimates that correlated above 0.75 with those from the full sample in over 95% of the bootstrapped samples. Taken as a set, these analyses suggested that the current sample size afforded reasonable accuracy and stability within the network findings.

#### 3.2.3. Unique, Proximal Links That Emerged

Network analysis using Gaussian graphical models is designed to uncover the most robust unique links amongst a set of correlated constructs. It does this by calculating the partial correlations between each pair of variables after controlling for every other variable in the analysis. The resulting partialized correlations are called edge-weights in network analysis. While this causes a majority of the pairwise associations to shrink to exceedingly small estimates near zero (which are then set to zero via LASSO regularization), this process allows the most proximal and unique links between pairs of variables to emerge, uncovering a deeper pattern of findings formerly obscured in a sea of moderate correlations. The results of this process are demonstrated in Figure 4 which shows the original zero-order correlation matrices for each model (below the diagonals) and the resulting simplified matrices of edge-weights resulting from the network analyses (above the diagonals).

#### 3.2.4. Interpreting Network Graphs

**Figure 5:**
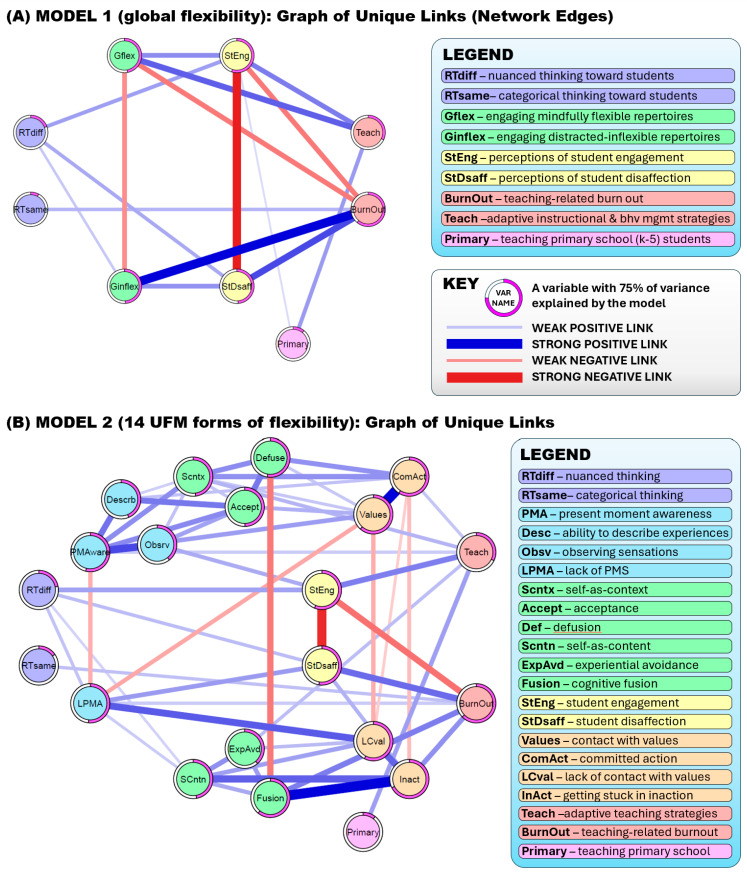
Network Graphs and Centrality Estimates. NOTE: The graphing of notably small edge weights (<0.04) has been suppressed in these graphs to aid interpretation by reducing visual noise (as those edge weights account for only nominal unique covariance).

To visualize these results, Figure 5A,B present network graphs for the two models. In these graphs, the variables (termed nodes in network analysis) are represented by circles and the proportion of variance accounted for in each variable is represented by the proportion of pink filling the ring around each node. As shown in the key of Figure 5, the unique associations that emerged (i.e., the edge-weights representing the partial correlations between each pair of variables after controlling for all of the other variables in the model) are represented as lines linking pairs of variables/nodes. The thicker and more deeply saturated lines represent stronger links, with blue lines indicating positive associations and red lines representing negative associations. The authors specified the spacing of the nodes in these graphs to facilitate interpretation by aligning their positions with the broader UFM conceptual framework (see Figure 1).

#### 3.2.5. Model 1

Focusing primarily on the strongest unique associations to emerge (i.e., the darker and thicker lines in Figure 5A—representing the largest amounts of unique shared variance), greater evidence of nuanced relational thinking toward students (i.e., seeing positive and negative students traits as more similar—labeled RTdiff in the graph) demonstrated robust unique links to higher teacher perceptions of both student engagement and disaffection in their classrooms, suggestive of potentially greater sensitivity and nuance in those conscious student evaluations. In contrast, greater evidence of categorical relational thinking toward students (i.e., seeing all positive student traits as strongly similar and all negative traits as strongly similar) was linked to higher levels of teacher burnout. Consistent with expectations (see Figure 1), greater perceptions of student engagement were uniquely linked to greater use of adaptive behavior management and teaching strategies and to lower levels of teacher burnout. In contrast, greater teacher perceptions of student disaffection were linked to greater teacher burnout. Taken as a set, these findings suggest that relational frames assessed and scored at an individual level from a student-focused relational task demonstrated meaningful links to conscious perceptions of students and to teacher burnout. Notably, teachers instructing primary school aged children (grades k-5—a dichotomous variable included as a control) was linked to more frequent use of adaptive instructional and behavior management strategies and to slightly higher perceptions of student engagement.

Turning to the global assessments of mindful-flexibility and distracted-inflexibility, higher levels of teachers generally engaging the mindful-flexibility repertoires in their classrooms was uniquely linked to: (1) greater teacher perceptions of student engagement, (2) teachers using more adaptive behavior management and teaching strategies in those classrooms, and (3) lower levels of teacher burnout. In contrast, greater teacher engagement of distracted-inflexible behavioral repertoires in their classrooms was uniquely linked to: (1) greater perceptions of student disaffection and (2) greater teacher burnout. Taken as a set, these results suggest that teachers’ general engagement of the mindful-flexibility and distracted-inflexibility behavioral repertoires of the UFM model were uniquely linked to classroom dynamics.

#### 3.2.6. Model 2

Once again focusing on the more robust unique links (as those account for larger amounts of the shared variance), the results presented in Figure 5B were broadly supportive of the UFM model when applied to a classroom setting (compare with top right model in Figure 1). Thus, a mindfully-flexible adaptive cascade emerged represented by a density of robust edges arcing across the sets of mindful flexibility skills in the top half of the graph. Consistent with UFM model predictions, skills within each stage were robustly linked to one another and to skills in the one or two subsequent stages, highlighting the interconnected nature of mindful flexibility behavioral repertoires. More specifically, greater engagement of mindful lenses (the light blue nodes of describing thoughts & feelings, observing sensations, & present moment attentive awareness) showed robust positive links to one another and to correspondingly higher levels of the light green nodes of self as context, acceptance, and defusion representing effective decentering from difficult experiences. Engagement of decentering skills were linked to one another and, in turn, linked to higher levels of the light orange nodes of committed action and maintaining contact with values representing right-mindful and value-driven behavior. Consistent with expectations, higher engagement of value-driven behavioral repertoires in the classroom was linked to more adaptive behavior management and teaching strategies. Higher levels of observing the physical sensations and beauty in each moment within the classroom was the only dimension of psychological flexibility to show a fairly robust direct link to higher perceptions of student engagement.

A corresponding distracted-inflexible reactive cascade also emerged represented by a density of robust edges arcing across the sets of distracted-inflexible skills in the bottom half of the graph. Specifically, teachers’ greater engagement of a distracted and inattentive lens within the classroom was linked to greater defensive reactivity (light green nodes of self-as-context, experiential avoidance, and fusion) and strongly to a greater difficulty in maintaining day-to-day contact with deeper values. Greater engagement of defensive reactivity in response to difficult classroom experiences was linked to greater aimless and haphazard behavior (light orange nodes of losing contact with values and getting stuck in inaction), which, in turn, was linked to greater feelings of burnout.

Turning to the most robust links from dimensions of distracted-inflexibility to classroom dynamics, engagement in cognitive fusion (getting stuck in difficult thoughts, feelings, and experiences in the classroom) and inaction demonstrated the most robust links to teachers’ feelings of burnout. This suggested that mentally and behaviorally shutting down in response to the ongoing chaos of teaching a large and diverse class of students was particularly tied to experiencing burnout toward students and teaching in general. The dimension of lack of present moment awareness (i.e., engaging a generally distracted and inattentive lens to the present moment) was linked to greater perceptions of student disaffection.

#### 3.2.7. Centrality Estimates

Turning to the indices of centrality estimated for Model 2 (Supplemental Online Figure S3), the four dimensions of the UFM model representing value-driven behavior (contact with values and committed action) and aimless haphazard behavior (lack of contact with values and inaction) emerged as markedly central, both in terms of the overall strength of their unique links to other nodes/processes in the model and in terms of the high level of integration and possible influence existing within the nodes to which they are directly linked (i.e., their expected influence). This serves to highlight the fundamentally behavioral nature of the UFM and its links to classroom dynamics.

## 4. Discussion

Through the use of Relational Frame Theory, Relational Density Theory, and the Unified Flexibility and Mindfulness conceptual frameworks, this study applied a Contextual Behavioral Science lens to understanding critical elements of teachers’ SEC and the potential mechanics underlying classroom dynamics. Building on the promising findings of ACT and mindfulness-based interventions with teachers (e.g., Biglan et al., 2013; Emerson et al., 2017; Jennings et al., 2017; Paliliunas et al., 2023; Roeser et al., 2013), the study conducted a broader (Model 1) and a more fine-grained, process-focused (Model 2), exploratory analysis of how teachers’ relational thinking about students and their engagement of various mindfully flexible behavioral repertoires (and distracted-inflexible repertoires) might be linked to: (1) teachers’ perceptions of student engagement and disaffection, (2) their use of adaptive behavior management and teaching strategies, and (3) burnout.

### 4.1. Interpretation of Results

#### 4.1.1. Cross-Sectional Support of the UFM Model in Classrooms

At a broader level, the results offered tentative, correlational, cross-sectional support for the UFM model when applied to classroom dynamics. This extends previous work on the UFM (e.g., Rogge & Daks, 2021; Rogge et al., 2024; Parker et al., 2024; Rogge et al., 2026) into educational settings. If replicated and extended in future work, this course of research could provide a rich and detailed scaffolding that is directly linked to the various therapeutic metaphors, visualizations, and exercises developed for ACT and mindfulness-based interventions. Thus, the current findings offer an early step toward cultivating a deeper understanding of how teachers’ SEC could be enhanced and maintained.

#### 4.1.2. Cross-Sectional Links of Relational Thinking

As anticipated, the results uncovered unique cross-sectional, correlational links from teachers’ relational thinking patterns (assessed indirectly) to their perceptions of student engagement and disaffection (assessed with self-report). Unexpectedly, stronger nuanced relational thinking about students was uniquely linked not only to higher perceptions of student engagement (as anticipated) but also to higher perceptions of student disaffection, suggesting that such a nuanced frame could help teachers more clearly see a mixture of engagement and disaffection in the same students. As this is one of the first studies to have both (1) examined patterns of relational thinking at the level of individual teachers (assessed with a student-focused relational task) and (2) applied RDT to a classroom environment, this offers initial evidence supporting the validity of relational tasks for assessing the relational thinking patterns toward students. These findings build on recent pilot work that developed (and demonstrated initial cross-sectional, correlational, predictive validity for) a novel method of (1) focusing a relational task on teachers’ perceptions of student traits and (2) analyzing the relational task data at an individual level instead of a group level (Palmer, 2025). As this is the first peer-reviewed manuscript to evaluate the concurrent predictive validity of this new method, its results should be interpreted cautiously. Future research is needed to critically evaluate and robustly validate this new method. Acknowledging that caveat, the current findings offer preliminary insights into the potential for patterns of relational thinking to shape how teachers view their students.

#### 4.1.3. Cross-Sectional Links of Student Engagement

As anticipated, teachers’ perceptions of student engagement demonstrated unique cross-sectional, correlational links to their greater use of adaptive behavior management and teaching strategies and to lower levels of burnout in both Models 1 and 2. These results align with previous findings linking student engagement to teaching strategies (e.g., Fredricks et al., 2004; Pianta et al., 2012) and burnout (e.g., Jennings & Greenberg, 2009; Skaalvik & Skaalvik, 2010). However, perceptions of student engagement failed to show strong unique links to their greater engagement of the more general value-driven behavior skills of the UFM model. If replicated, this would begin to suggest that those links might be more specific and potentially somewhat independent of teachers’ more general engagement of value-driven behavior in the classroom.

#### 4.1.4. Cross-Sectional Links of Student Disaffection

Consistent with expectations, perceptions of student disaffection demonstrated unique cross-sectional, correlation links to teachers more often getting stuck in inaction in response to classroom chaos and to higher levels of burnout. However, after controlling for all other constructs in the model, teachers’ perceptions of student disaffection failed to show a unique cross-sectional link to behavior management and teaching strategies. This differs somewhat from previous literature on student disaffection (Fredricks et al., 2004; Pianta et al., 2012). If these initial cross-sectional, correlational findings are replicated and extended in future work, this would begin to suggest that by controlling for a more comprehensive set of classroom dynamics, a more nuanced picture could emerge, refining the scope of previous findings with this construct.

#### 4.1.5. Cross-Sectional Links of UFM Processes

In Model 1, teachers’ global engagement of the mindful-flexibility skills demonstrated cross-sectional, correlational links to greater perceptions of student engagement whereas teachers’ global engagement of the distracted-inflexible behavioral repertoires demonstrated a unique cross-sectional link to greater perceptions of student disaffection. The Model 2 results clarified these findings, as the only mindful-flexibility process showing a unique cross-sectional link to greater perceptions of student engagement (after controlling for all other variables in the model) was observing sensations in the classroom. Similarly, the only distracted-reactive-inflexible processes showing cross-sectional links to greater perceptions of student disaffection were lack of present moment awareness and getting stuck in inaction in the classroom setting. These cross-sectional results, if replicated in future work, would begin to suggest that although teachers’ engagement of mindful-flexibility behavioral repertoires in the classroom might be more notably linked to their own teaching strategies and well-being (e.g., burnout), engaging specific UMF behavioral repertoires might be more moderately linked to teachers’ perceptions of students.

### 4.2. Implications

#### 4.2.1. Novel Conceptual Integration

The present study offers a novel integration of Relational Density Theory (RDT; Belisle & Dixon, 2020) and the Unified Flexibility and Mindfulness model (UFM; Rogge & Daks, 2021) by examining how the organization of teachers’ relational networks may scaffold adaptive versus maladaptive patterns of classroom functioning. Whereas RDT has almost exclusively been applied at a group-level (characterizing the density and nature of relational networks of groups in laboratory settings), the UFM model has been applied at an individual level (exploring possible mechanistic chains linking various mindful-flexibility processes that are triggered in response to difficult thoughts, feelings, and experiences in the classroom). Although both are grounded within the broader CBS conceptual framework, these traditions have developed in parallel with little integration. The current work begins to bridge this gap by conceptualizing relational thinking as a structural substrate that could shape classroom dynamics. Specifically, densely clustered networks of student descriptors may reflect or promote categorical, rigid modes of thinking, which in turn may amplify the use of defensive-reactive-inflexible processes and shape teachers’ perceptions of students, use of behavior management and teaching strategies, and increasing frustrations in the classroom. By situating teachers’ relational thinking toward students within a broader process model of flexibility and mindfulness, this study begins to extend both frameworks and provides a more comprehensive and contextualized account of the cognitive, behavioral, and affective mechanisms underlying teacher burnout, instructional practices, and classroom climate.

#### 4.2.2. Relational Thinking

Unexpectedly, both Model 1 and 2 uncovered a moderate link from higher categorical relational thinking to higher levels of burnout, highlighting unique risks associated with such thinking. With the exception of that unique link, patterns of relational thinking were primarily linked to the outcomes of teaching strategies and burnout indirectly via their robust links to perceptions of student engagement and disengagement. If replicated in future studies, these findings could help to contextualize the robust finding in the literature linking perceptions of student engagement and disengagement to teaching strategies and burnout (see Fredricks et al., 2004 for a review) by highlighting perceptions of students as an intermediate mechanism by which relational thinking toward students might shape the classroom environment. If replicated by future work, this would begin to suggest that the ability to detect and perceive student engagement, even in students with challenging behavior, could potentially serve as a major source of resilience for teachers, encouraging them to use more adaptive approaches to behavior management and teaching and possibly even protecting them from burnout.

#### 4.2.3. Mindful-Flexibility

Another unique contribution of the current study was uncovering links from global mindful-flexibility (and distracted-inflexibility) to classroom strategies and teacher burnout in Model 1. This builds on the work demonstrating that ACT-based and mindfulness-based interventions can be effective at improving teachers’ well-being and teaching styles (e.g., Biglan et al., 2013; Emerson et al., 2017) by highlighting the positive and negative cascades of behavioral repertoires within the UFM model as possible mechanisms. The findings of Model 2 extended that work further by highlighting specific UFM processes demonstrating links to classroom strategies and burnout. Notably, one process from each of the stages of the positive cascade within the UFM model emerged with unique links to the use of adaptive behavior management and teaching strategies: present moment awareness (i.e., living fully in each moment) from the mindful lenses stage, self-as-context (i.e., maintaining a broader perspective even in the face of disruptions and challenging student behavior) from the decentering stage, and committed action (i.e., continuing to take meaningful steps toward your deeper goals despite the stresses, strains, setbacks, and distractions of day-to-day life in a classroom) from the value-driven behavior stage. If these findings continue to emerge in future studies, those skills might represent particular sources of resilience for teachers.

#### 4.2.4. Distracted-Inflexibility

Two distracted-reactive-inflexible processes emerged with unique links to burnout: (1) fusion (i.e., having negative thoughts and experiences continually run through one’s mind like a broken record) from the defensively reacting stage of the UFM negative cascade and (2) getting stuck in inaction (i.e., negative thoughts and experiences effectively stalling out teachers’ progress toward deeper goals for themselves and their students) from the aimless and haphazard behavior stage. These findings begin to suggest that those processes might represent key risk areas. If future work continues to highlight these same specific processes as crucial, they could even serve as potential targets for intervention to prevent and treat teacher burnout.

### 4.3. Limitations

Despite the notable conceptual and substantive contributions of the current study, a number of factors limit the generalizability of its findings.

First, building on recent pilot work (Palmer, 2025), this is the first peer-reviewed study to develop and validate both this specific Relational Task (teachers responding to a set of student-focused adjectives) as well as a new method of analyzing Relational Task data that allows for scoring at the level of individual teachers (as opposed to the group-level analyses that have predominated the RDT literature; e.g., Belisle & Dixon, 2020; Hutchison et al., 2023). The results offered initial support and validation of this approach by: (1) presenting item-level EFA results supporting the teacher-level scoring of the relational task (Supplemental Online Tables S1 and S2), (2) demonstrating discriminant validity for this new approach (within the correlations presented in Figure 4), and (3) demonstrating unique concurrent predictive validity of this approach—linking the relational thinking of individual teachers to various aspects of classroom dynamics and teacher burnout within the network analysis results (Models 1 and 2; Figure 4 and Figure 5). However, the results based on this new approach should still be interpreted with caution. Future research should seek to replicate and extend the current findings to further refine and validate this novel approach.

Second, the study was entirely cross-sectional and so, directions of causality could not be determined. Although the UFM model posits reciprocal directions of influence between the variables examined, future work should make use of multi-wave designs that can support cross-lagged predictive analyses to more directly assess potential directions of influence within the links that emerged in these analyses. Future work could also extend the current findings with experimental designs and randomized controlled trials of potential interventions to strengthen teacher SEC and resilience.

Third, the sample was 79% female and 85% white with an average age of 42. Although this reasonably mirrors the demographics of teachers in the US (77% female, 80% white, with an average age of 43; see Schaeffer, 2024), the lower representation of male and minority teachers in the current sample raises concerns about the generalizability of these findings. Future work should seek larger samples, potentially oversampling male and minority teachers, to allow the generalizability of the current findings to be directly tested in those smaller demographic groups. Extending this point, the sample collected data entirely online which might have served to limit diversity in the final sample. Counterbalancing this concern, recent national data suggests notable declines the technology gap due in large part to the rapid permeation of smart phones into the lives of individuals in the US (Pew Research, 2021), thereby making internet access increasingly available to individuals from all walks of life. In addition, the demographics of the current sample closely mirror demographics of teachers within the United States, suggesting that the sample was reasonably representative. However, to more directly address these concerns, future studies of the UFM model in classrooms should seek samples with greater diversity to ensure these findings continue to hold across all demographic groups.

Fourth, the innovative approach of the current study required slight modifications of existing measures (e.g., the CBI, the UFM) to apply them to new contexts and focus them on new targets. The study sought to ameliorate these concerns by: (1) presenting item-level CFA results supporting the factor structures of the modified CBI and UFM scales (Supplemental Online Tables S3 and S4), (2) presenting evidence of their discriminant validity in the correlations presented in Figure 4, and (3) presenting initial evidence of their concurrent predictive validities within the network analysis results (Models 1 and 2; Figure 5). In addition, a number of previous studies (across multiple languages) have validated teacher-focused versions of the Copenhagen Burnout Inventory using student-related burnout items (e.g., Fiorilli et al., 2015; Milfont et al., 2008; Piperac et al., 2021). However, as this was the first study to make use of these specific modified assessments, the results should be interpreted cautiously. Future work replicating and extending the current findings is needed to fully validate these modified CBI and UFM measures.

Fifth, the current study focused on teachers’ experiences, perceptions, behaviors, and outcomes in this first attempt to inform our understanding of SEC via the UFM model. Given that focus, the study did not examine how these various factors might impact student learning and academic achievement. Thus, future work could meaningfully extend the current findings by collecting data on academic achievement to explore links between the constructs examined in this study and academic success. Extending this point, future research could meaningfully build on this work by collecting data on the classroom and instructional dynamics from students to augment the information collected by teachers.

Sixth, the current study focused at the level of individual teachers and how they navigate the day-to-day challenges they face. Indeed, the current findings suggest that, from that individual perspective, RDT and the UFM conceptual framework might offer key insights in understanding and promoting greater SEC and resilience among teachers. However, there is a growing field of work highlighting the power of a positive school climate—at the collective level—to promote students’ healthy development and academic achievement, and teacher retention (Cohen et al., 2009). In fact, collective-level contextual and social factors have been linked to greater teacher well-being (Hascher & Waber, 2021). Consistent with this work, the construct of collective teacher efficacy has been linked to greater academic achievement (Tschannen-Moran & Barr, 2004). Such findings have led researchers to conceptualize schools as collective systems in which group-level factors like teachers’ trust in students and parents, their collective efficacy, and the schools’ drive for academic excellence can create a collective “academic optimism” that holds the potential to fuel student achievement (Hoy et al., 2006). Thus, future work could augment the current findings by including group and structural level constructs like administrative support, collective teacher efficacy, and academic optimism to identify additional sources of resilience and intervention for teachers and schools.

Finally, the UFM model incorporates 14 distinct behavioral repertoires into a larger 3-stage process framework. If the current findings are replicated in future studies, the UFM model (and its grounding in 20+ years of intervention research) could hold tremendous potential for enriching work on understanding and promoting teacher SEC. However, future work on the UFM model could be enhanced by considering the inclusion of additional behavioral processes that have been robustly linked to healthy relationships and individual well-being (e.g., non-attachment, self-compassion, emotion regulation skills).

### 4.4. Conclusions

Despite its limitations, the current study offers a cross-sectional and correlational first-look into how Contextual Behavioral Science might inform our understanding of classroom dynamics and teacher well-being. By integrating a novel individual-focused approach to Relational Density Theory within the broader scaffolding of the Unified Flexibility and Mindfulness model, the study takes the first steps toward contextualizing our understanding of classroom dynamics and teachers’ SEC within the broader CBS framework. It is important to note that the current findings should be interpreted cautiously as the study represents one of the first efforts to conceptually integrate the fields of CBS and teacher SEC research. However, if these findings continue to replicate across a more diverse range of samples and study designs (e.g., longitudinal studies, experimental designs, randomized controlled trials of interventions), this program of research could hold the potential to offer key insights into the specific mechanisms that might serve as optimal points of risk and resilience for teachers, eventually identifying highly effective intervention targets to promote and maintain teachers’ SEC and well-being.

## Data Availability

All study materials have been made available on the second author’s osf.io profile under the “The Understanding Teachers’ Perspectives Survey” project (https://osf.io/73z6d, accessed on 27 May 2026). The preregistration for the project and this manuscript is also available in that project, as are the SPSS 29.0 syntax and R version 4.3.2 (2023-10-31 ucrt) syntax and relevant output. Finally, the data is available upon reasonable request within a component of that same osf.io project.

## Supplementary Materials

The following supporting information can be downloaded at: https://www.mdpi.com/article/10.3390/bs16061018/s1, Table S1. EFA Factor Loadings of Positive-Negative Adjective Pairs on the Relational Task Used to Represent More Nuanced Thinking. Table S2. EFA Factor Loadings of Positive–Positive & Negative–Negative Adjective Pairs on the Relational Task Used to Represent More Categorical Thinking. Table S3. CFA Standardized Loadings of the Items of the UFM on Their Respective Subscales. Table S4. CFA Standardized Loadings of the Items of the CBI Work-Related Burnout and Student-Related Burnout Subscales on Their Respective Latent Factors. Figure S1. Simulations for detecting observed networks at different sample sizes. Figure S2. Bootstrapping results to evaluate stability/precision of network findings. Figure S3. Centrality Coefficients from Each Model.
