# Supplementary material for: Flexible Teachers, Thriving Classrooms: A Unified Flexibility and Mindfulness (UFM) Model of Classroom Dynamics, Teaching Practices, and Teacher Burnout
Source: Behav Sci (Basel). 2026 Jun 17;16(6):1018. doi: 10.3390/bs16061018 (PMC13296330; doi:10.3390/bs16061018)

Supplemental Online Table S1. *EFA Factor Loadings of Positive-Negative Adjective Pairs on the Relational Task Used to Represent More Nuanced Thinking*

**COMPONENT OF RELATIONAL TASK**				
	Specific text	Factor Loadings	Specific text	Factor Loadings
**STEM**				
	STUDENT TRAITS: In your experience, how closely related are these two student characteristics?			
**ADJECTIVE PAIRS**				
	Distracted-Cooperative	**.81**	Loud-Thoughtful	**.74**
	Disrespectful-Focused	**.78**	Loud-Cooperative	**.73**
	Defiant-Focused	**.78**	Angry-Respectful	**.72**
	Angry-Thoughtful	**.77**	Loud-Successful	**.72**
	Disrespectful-Successful	**.77**	Unsuccessful-Respectful	**.72**
	Unsuccessful-Focused	**.77**	Unsuccessful-Cooperative	**.68**
	Angry-Successful	**.77**	Unsuccessful-Thoughtful	**.67**
	Defiant-Successful	**.77**	Defiant-Cooperative	**.66**
	Disrespectful-Thoughtful	**.76**	Disrespectful-Quiet	**.65**
	Loud-Focused	**.76**	Disrespectful-Respectful	**.65**
	Disrespectful-Cooperative	**.76**	Loud-Quiet	**.64**
	Defiant-Thoughtful	**.75**	Unsuccessful-Successful	**.63**
	Angry-Focused	**.75**	Distracted-Thoughtful	**.59**
	Loud-Respectful	**.75**	Defiant-Quiet	**.58**
	Angry-Cooperative	**.74**	Unsuccessful-Quiet	**.57**
	Distracted-Respectful	**.74**	Angry-Quiet	**.54**
	Distracted-Successful	**.74**	Distracted-Quiet	**.50**

NOTE: The items of the Relational Task were presented on a 10-point scale. This table presents the results of an EFA using Principle Axis Factoring extraction to extract the single dominant factor emerging in these 34 items (accounting for 51.6% of the variance).

Supplemental Online Table S2. *EFA Factor Loadings of Positive-Positive & Negative-Negative Adjective Pairs on the Relational Task Used to Represent More Categorical Thinking*

**COMPONENT OF RELATIONAL TASK**				
	Specific text	Factor Loadings	Specific text	Factor Loadings
**STEM**				
	STUDENT TRAITS: In your experience, how closely related are these two student characteristics?			
**ADJECTIVE PAIRS**				
	Respectful-Successful	**.74**	Angry-Disrespectful	**.62**
	Cooperative-Focused	**.73**	Quiet-Successful	**.61**
	Focused-Successful	**.72**	Thoughtful-Quiet	**.61**
	Cooperative-Successful	**.71**	Defiant-Distracted	**.60**
	Thoughtful-Successful	**.69**	Angry-Unsuccessful	**.59**
	Respectful-Focused	**.69**	Disrespectful-Distracted	**.59**
	Thoughtful-Respectful	**.67**	Distracted-Loud	**.59**
	Cooperative-Respectful	**.66**	Angry-Loud	**.59**
	Respectful-Quiet	**.66**	Distracted-Unsuccessful	**.58**
	Cooperative-Quiet	**.66**	Defiant-Unsuccessful	**.57**
	Focused-Quiet	**.65**	Angry-Defiant	**.56**
	Defiant-Loud	**.65**	Disrespectful-Loud	**.55**
	Thoughtful-Cooperative	**.65**	Angry-Distracted	**.53**
	Disrespectful-Unsuccessful	**.65**	Loud-Unsuccessful	**.52**
	Thoughtful-Focused	**.62**		

NOTE: The items of the Relational Task were presented on a 10-point scale. This table presents the results of an EFA using Principle Axis Factoring extraction to extract the single dominant factor emerging in these 29 items (accounting for 42.1% of the variance).

Supplemental Online Table S3. *CFA Standardized Loadings of the Items of the UFM on their Respective Subscales*

COMPONENT / SUBSCALE OF UFM<br>Specific stem / item text	Est	SE	Est/SE	p
**STEM**				
IN THE LAST 2 WEEKS, when I was in my classroom with students…				
**PRESENT MOMENT ATTENTIVE AWARENESS (a rudimentary form of mindfulness)**				
I paid close attention to what I was thinking and feeling	**.89**	.014	65.295	<.001
I was attentive and aware of my emotions	**.92**	.011	85.686	<.001
I was in touch with the ebb and flow of my thoughts and feelings	**.94**	.008	117.969	<.001
I was in tune with my thoughts and feelings from moment to moment	**.93**	.009	100.028	<.001
I strived to remain mindful and aware of my own thoughts and emotions	**.88**	.014	62.092	<.001
**DESCRIBING THOUGHTS/FEELINGS**				
I was good at finding the words to describe my feelings	**.90**	.014	64.086	<.001
I was easily able to put my beliefs, opinions, and expectations into words	**.90**	.014	65.391	<.001
Even when I was feeling terribly upset, I found a way to put it into words	**.80**	.023	34.255	<.001
My natural tendency was to put my experiences into words	**.77**	.025	30.169	<.001
I could usually describe how I felt at the moment in considerable detail	**.89**	.014	63.219	<.001
**OBSERVING SENSATIONS**				
I paid attention to sensations, such as the air on my face or the sun on my skin	**.84**	.023	37.121	<.001
I noticed visual elements in art or nature, such as colors, shapes, textures, or patterns of light and shadow	**.86**	.021	40.392	<.001
I stayed alert to the sensations of my day-to-day classroom life	**.67**	.035	19.052	<.001
I paid attention to sounds such as clocks ticking, birds chirping, or cars passing	**.73**	.031	23.581	<.001
I paid attention to how my emotions affected my thoughts and behavior	**.59**	.041	14.403	<.001
**ACCEPTANCE (being open and accepting of all experiences, even the difficult or challening ones)**				
I made room to fully experience negative thoughts and emotions, breathing them in rather than pushing them away	**.87**	.016	54.208	<.001
When I had an upsetting thought or emotion, I tried to give it space rather than ignoring it	**.90**	.014	63.279	<.001
I was receptive to observing unpleasant thoughts and feelings without interfering with them	**.83**	.020	41.018	<.001
I tried to make peace with my negative thoughts and feelings rather than resisting them	**.86**	.018	47.996	<.001
I opened myself to all of my feelings, the good and the bad	**.83**	.020	42.051	<.001
**SELF-AS-CONTEXT (maintaining a broader perspective even in the face of challenges and setbacks)**				
I tried to keep perspective even when things knocked me down	**.86**	.017	50.891	<.001
When I was scared or afraid, I still tried to see the larger picture	**.85**	.018	46.135	<.001
Even when I felt hurt or upset, I tried to maintain a broader perspective	**.89**	.014	61.588	<.001
When something painful happened, I tried to take a balanced view of the situation	**.88**	.016	56.463	<.001
I carried myself through tough moments by seeing my life from a larger viewpoint	**.83**	.020	40.688	<.001
**DEFUSION (experiencing difficult thoughts and feelings gently without clinging to them)**				
I was able to let negative feelings come and go without getting caught up in them	**.89**	.015	59.526	<.001
When I was upset, I was able to let those negative feelings pass through me without clinging to them.	**.91**	.013	69.988	<.001
When I was scared or afraid, I was able to gently experience those feelings, allowing them to pass.	**.72**	.030	24.245	<.001
I was able to step back and notice negative thoughts and feelings without reacting to them	**.85**	.018	45.951	<.001
In tough situations, I was able to notice my thoughts and feelings without getting overwhelmed by them	**.85**	.019	45.726	<.001

Supplemental Online Table S3 continued. *CFA of the UFM*

COMPONENT / SUBSCALE OF UFM<br>Specific stem / item text	Est	SE	Est/SE	p
**CONTACT WITH VALUES (maintaining contact with your deeper values on a daily basis and allowing them to guide you)**				
I was very in touch with what is important to me and my life	**.89**	.015	59.351	<.001
I tried to connect with what is truly important to me on a daily basis	**.88**	.015	57.98	<.001
I stuck to my deeper priorities in life	**.83**	.021	40.066	<.001
Even when it meant making tough choices, I still tried to prioritize the things that were important to me	**.85**	.018	46.333	<.001
My deeper values consistently gave direction to my life	**.81**	.022	37.257	<.001
**COMMITED ACTION (taking steps toward your deeper goals even in the midst of challenges and setbacks)**				
Even when I stumbled in my efforts, I didn't quit working toward what is important	**.89**	.013	66.212	<.001
Even when things got tough, I was still able to take steps toward what I value in life.	**.93**	.010	94.443	<.001
Even when things got stressful and hectic, I still worked toward things that were important to me	**.87**	.016	53.606	<.001
I didn't let setbacks slow me down in taking action toward what I really want in life	**.85**	.017	48.776	<.001
I didn't let my own fears and doubts get in the way of taking action toward my goals	**.89**	.014	63.913	<.001
**EXPERIENTIAL AVOIDANCE (avoiding and distracting yourself from difficult experiences rather than facing them)**				
When I had a bad memory, I tried to distract myself to make it go away	**.88**	.015	60.132	<.001
When unpleasant memories came to me, I tried to put them out of my mind	**.91**	.012	72.418	<.001
I tried to distract myself when I felt unpleasant emotions	**.87**	.016	55.27	<.001
When something upsetting came up, I tried very hard to stop thinking about it	**.89**	.014	61.947	<.001
If there was something I didn't want to think about, I would try many things to get it out of my mind	**.90**	.013	70.467	<.001
**LACK OF PRESENT MOMENT AWARENESS (a distracted an inattentive approach to daily life)**				
I did most things on "automatic" with little awareness of what I was doing	**.89**	.014	64.884	<.001
I went through most days without paying much attention to what I was thinking or feeling	**.78**	.024	32.447	<.001
I floated through most days without paying much attention	**.90**	.013	67.441	<.001
I did most things mindlessly without paying much attention	**.89**	.014	63.02	<.001
Most of the time, I was just going through the motions without paying much attention	**.90**	.013	67.732	<.001
**SELF-AS-CONTENT (judging and shaming yourself for having difficult thoughts, feelings, and experiences)**				
I thought some of my emotions were bad or inappropriate and I shouldn't feel them	**.90**	.012	72.331	<.001
I believed some of my thoughts are abnormal or bad and I shouldn't think that way	**.88**	.014	61.984	<.001
I told myself that I shouldn't be feeling the way I'm feeling	**.93**	.010	92.23	<.001
I told myself I shouldn't be thinking the way I was thinking	**.91**	.012	77.762	<.001
I criticized myself for having irrational or inappropriate emotions	**.91**	.011	79.954	<.001
**COGNITIVE FUSION (clinging to difficult thoughts and feelings so that they continuously run around in your mind)**				
Negative thoughts and feelings tended to stick with me for a long time	**.92**	.011	83.798	<.001
Distressing thoughts tended to spin around in my mind like a broken record	**.90**	.012	72.246	<.001
It was very easy to get trapped into unwanted thoughts and feelings	**.89**	.013	68.029	<.001
When I had negative thoughts or feelings, it was very hard to see past them	**.87**	.015	57.68	<.001
When something bad happened, it was hard for me to stop thinking about it	**.90**	.013	68.748	<.001

Supplemental Online Table S3 continued. *CFA of the UFM*

COMPONENT / SUBSCALE OF UFM				
Specific stem / item text	Est	SE	Est/SE	p
**LACK OF CONTACT WITH VALUES (easily growing distracted from your deeper goals and values by the stresses of life)**				
My priorities and values often fell by the wayside in my day-to-day life	**.87**	.016	52.919	<.001
The things that I value the most often fell off my priority list completely	**.86**	.017	49.666	<.001
When life got hectic, I often lost touch with the things I value	**.90**	.014	65.938	<.001
I didn't usually have time to focus on the things that are really important to me	**.81**	.022	36.275	<.001
When times got tough, it was easy to forget about what I truly value	**.84**	.019	43.102	<.001
**GETTING STUCK IN INACTION (allowing difficult thoughts/feelings to derail you from working toward your deeper goals)**				
Negative feelings often trapped me in inaction	**.90**	.014	64.889	<.001
Getting upset left me stuck and inactive	**.87**	.016	52.706	<.001
Negative feelings easily stalled out my plans	**.88**	.015	59.744	<.001
Negative experiences derailed me from what's really important	**.84**	.019	43.091	<.001
Unpleasant thoughts and feelings easily overwhelmed my efforts to deepen my life	**.84**	.019	45.497	<.001

NOTE: The 70 items of the UFM were not modified for the current study but employed a stem that focused them specifically on the behavioral repertoires teachers engage in response to difficult and challenging thoughts, feelings, and experiences within their classrooms when interacting with students ("***IN THE LAST 2 WEEKS, when I was in my classroom with students...*** "). All UFM items were presented with a 6-point response scale (***1=Never, 2=Rarely, 3=Occasionally, 4=Often, 5=Very often, 6=All of the time***). This table presents the standardized item loadings and fit results of a CFA (run in Mplus) testing the 14-factor solution. As shown below, the 14-factor model demonstrated adequate fit in the current sample, suggesting that applying the UFM scale to teachers' behavior in the classroom yielded responses that continued to support the 14 subscale factor structure of the UFM.

FIT INDICES:	
**14-Factor Model**	**Adequate Fit Thresholds**
$\chi^2(2254)$ = **3781**, $p < .001$	
**CFI = .929**	**above .90**
**RMSEA = .047, 90% CI: LL = .044, UL = .050**	**below .08**
**SRMR = .049**	**below .10**

Supplemental Online Table S4. *CFA Standardized Loadings of the Items of the CBI Work-Related Burnout and Student-Related Burnout Subscales on Their Respective Latent Factors*

COMPONENT / SUBSCALE OF UFM				
Specific stem / item text	Est	SE	Est/SE	p
**STEM**				
IN THE LAST 2 WEEKS, when I was in my classroom with students...				
**WORK-RELATED BURNOUT**				
Was your work emotionally exhausting?	**.85**	.018	46.956	<.001
Did you feel burned out because of your work?	**.92**	.012	79.152	<.001
Did you your work frustrate you?	**.83**	.019	43.029	<.001
Were you exhausted in the morning at the thought of another day at work?	**.88**	.015	59.807	<.001
Did you feel that every working hour was tiring for you?	**.89**	.014	64.591	<.001
Did you have enough energy for family and friends during leisure time?	**-.47**	.046	-10.285	<.001
**STUDENT-RELATED BURNOUT**				
Did you find it hard to work with students?	**.86**	.018	48.154	<.001
Did you find it frustrating to work with students?	**.85**	.018	47.328	<.001
Did it drain your energy to work with students?	**.85**	.018	47.673	<.001
Did you feel that you gave more than you got back when you worked with students?	**.75**	.027	27.779	<.001
Were you tired of working with students?	**.90**	.013	69.162	<.001
Did you sometimes wonder how long you would be able to continue working with students?	**.84**	.019	44.239	<.001
**CORRELATION BETWEEN LATENT FACTORS**				
BO-Work with BO-Student	**.84**	.020	41.218	<.001

NOTE: The 6 items of the CBI work-related burnout subscale that we used were not modified for the current study but employed a stem that focused them specifically on feelings of burnout toward teaching (as all respondents were working as teachers; "***IN THE LAST 2 WEEKS< when I was in my classroom with students...*** "). The 6 items of the CBI patient-related burnout scale were modified slightly by replacing the word "***patients*** " with the word "***students*** " to help focus the items on burnout related to the classroom. All CBI items were presented with a 6-point response scale (***1=Never, 2=Rarely, 3=Occasionally, 4=Often, 5=Very often, 6=All of the time***). This table presents the standardized item loadings and fit results of a CFA (run in Mplus) testing the fit of the expected two-factor solution. As shown below, the two-factor model demonstrated adequate fit whereas a one-factor model failed to demonstrate adequate fit. Qualifying the fit of the two-factor solution, the CFA results revealed an extremely high correlation between the two latent factors (r = .84), suggesting that they were largely assessing a common construct (as they shared over 70% of their variance). Given that markedly robust correlation between the latent factors (which corresponded to a robust correlation between subscale scores), scores on the two subscales were averaged to create an overall burnout score to be used in the network analyses - thereby helping to reduce excessive collinearity in the model.

FIT INDICES:

Two-Factor Model	One-Factor Model	Adequate Fit Thresholds
$\chi^2(50)$ = **146**, p < .001	$\chi^2(52)$ = **415**, p < .001	
**CFI = .972**	**CFI = .892**	**above .90**
**RMSEA = .079, 90% CI: LL = .065, UL = .094**	**RMSEA = .153, 90% CI: LL = .140, UL = .167**	**below .08**
**SRMR = .034**	**SRMR = .054**	**below .10**

Supplemental Online Figure S1. *Simulations for detecting observed networks at different sample sizes.*

Correlations of edge weights between observed and simulated networks along with the sensitivity and specificity for detecting those edges at different sample sizes.

A) From simulations of Model 1

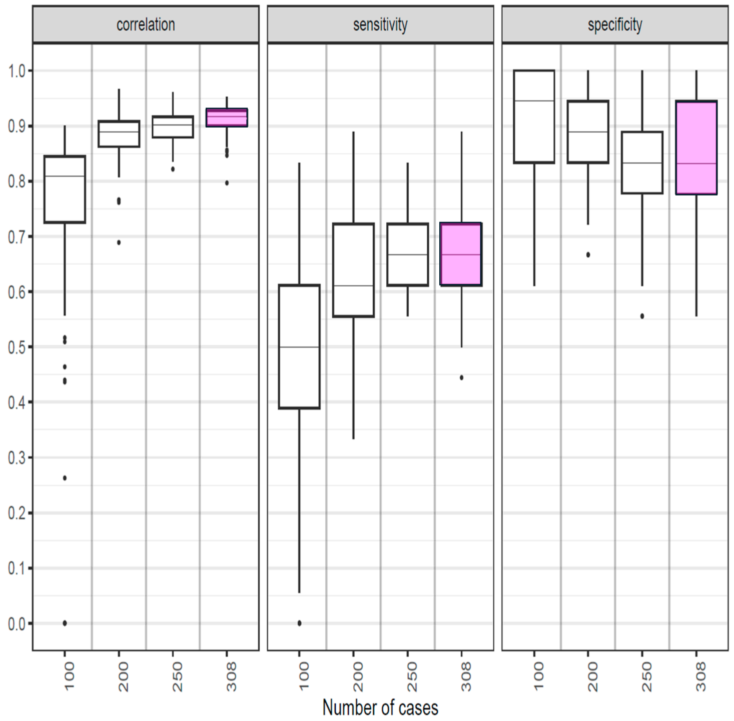

B) From simulations of Model 2

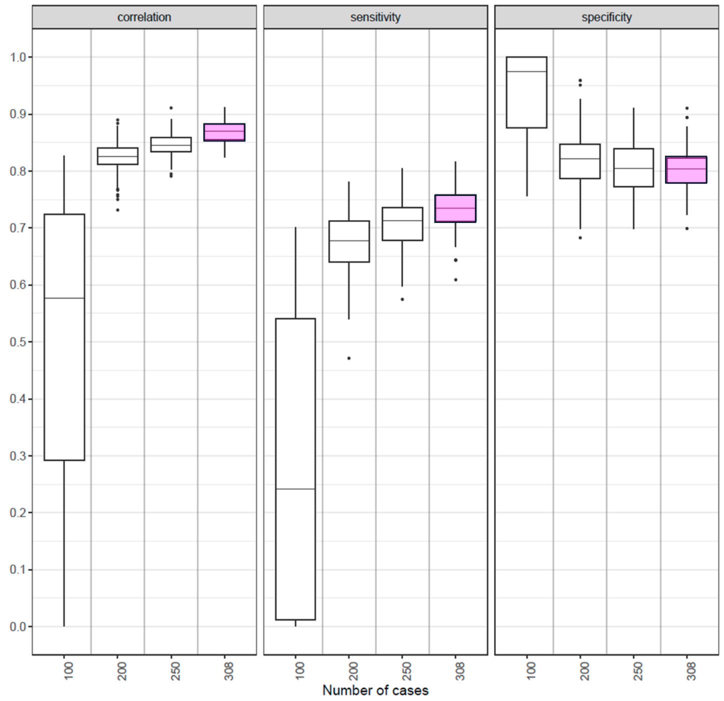

Correlations of centrality indices between observed and simulated networks at different sample sizes.

C) From simulations of Model 1

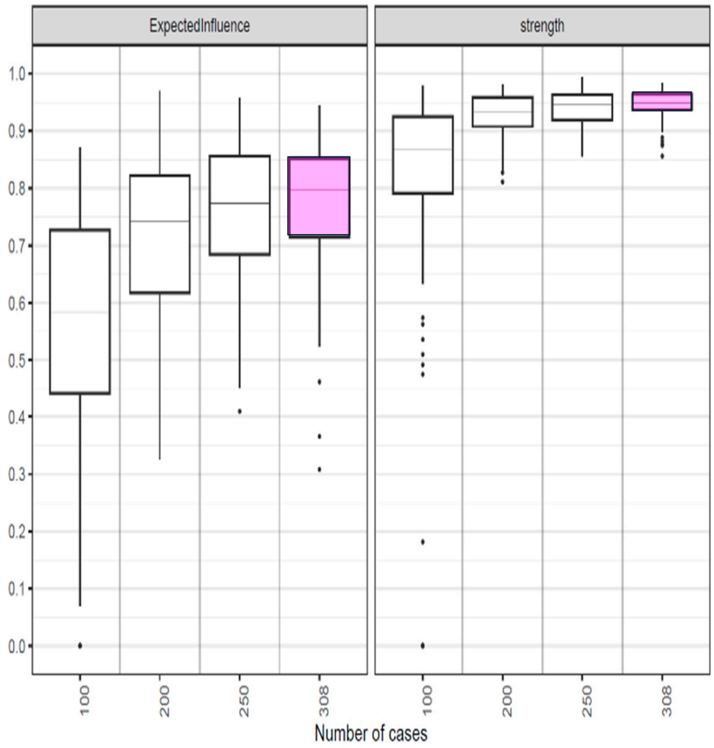

D) From simulations of Model 2

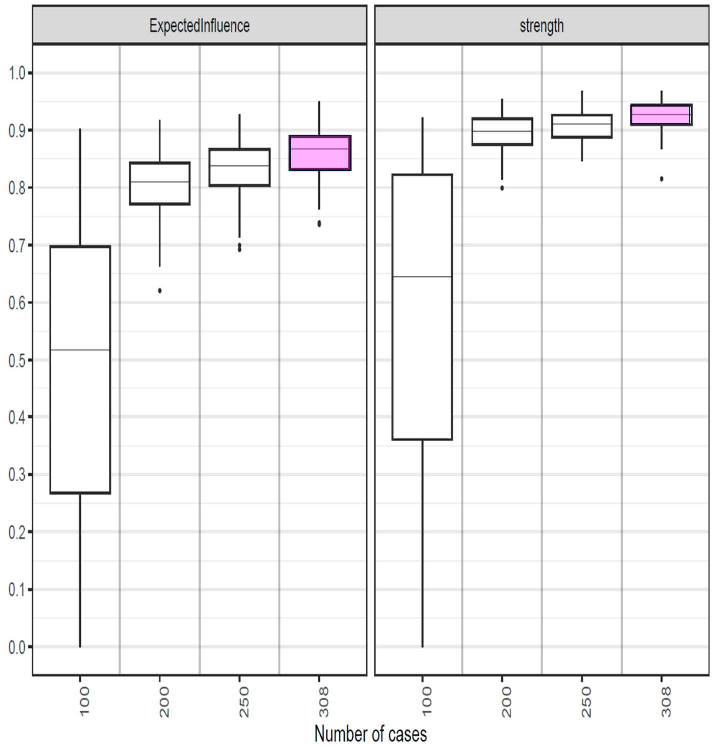

Narrative Supplemental Online Figure S1:

Following current best practices (e.g., Epskamp & Fried, 2018), we used the netSimulator function within the bootnet package for R (Epskamp & Fried, 2021) to compare the results obtained within our data to results from 1000 simulations across a number of sample sizes.

PANELS A and B: The results obtained from simulated datasets demonstrated robust correlations with the edge weight estimates from the actual data (average correlations of .91for Model 1 and .80 for Model 2 within samples of 308 subjects – see pink boxes). These findings suggest reasonably high levels of stability for the current findings. In addition, the simulation results revealed fairly high levels of specificity for both models (.80 to .85 on average, suggesting reasonably high confidence in the edges that emerged) and adequate sensitivity for detecting those edges.

PANELS C and D: Extending this, the centrality estimates for closeness, expected influence, and strength (see Supplemental Online Figures S1C and S1D) also demonstrated robust correlations with the corresponding centrality estimates from the actual data (correlations above .80 for samples of 308 subjects), suggesting similar stability for those centrality findings for each model.

Supplemental Online Figure S2. *Bootstrapping results to evaluate stability / precision of network findings.*

Bootstrapped (non-parametric) confidence intervals for the edge weights estimated.

A) From Model 1

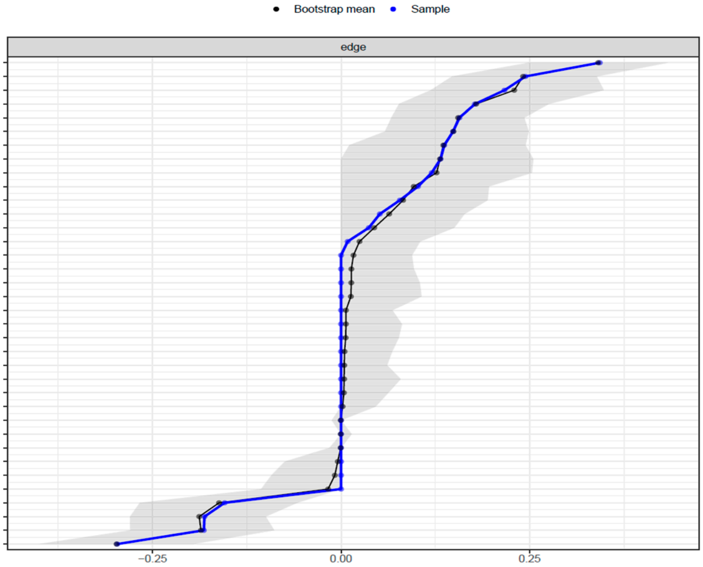

B) From Model 2

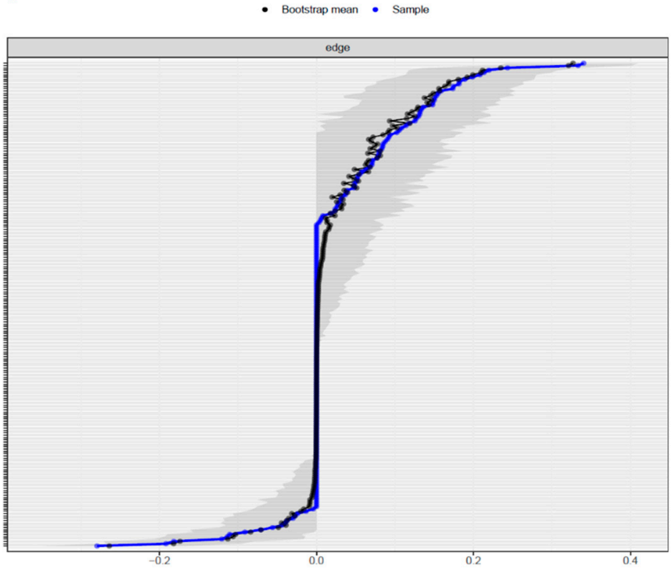

Stability of centrality indices using estimates from case-dropping bootstrapped samples.

C) From Model 1

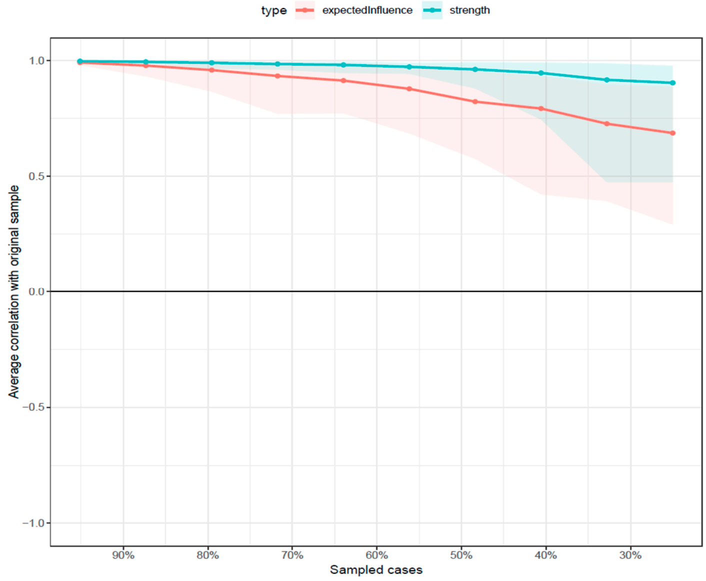

D) From Model 2

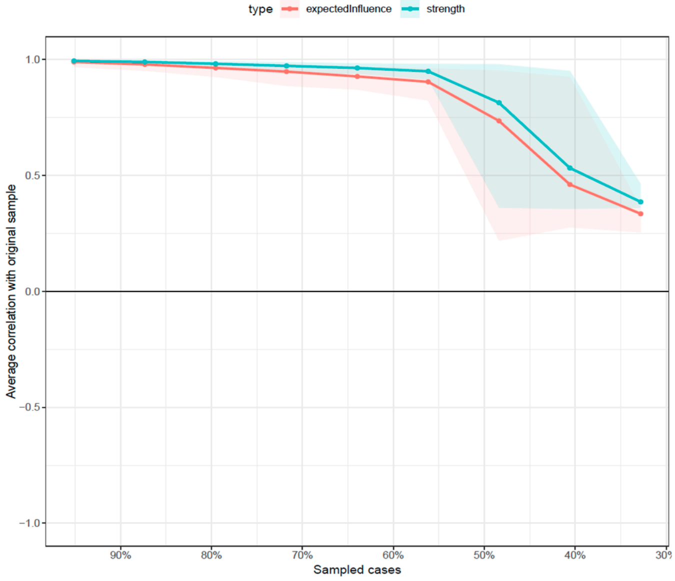

Narrative Supplemental Online Figure S2:

PANELS A and B: We also used the bootnet function of the bootnet package to generate 1000 nonparametric bootstrapped samples to obtain 95% confidence intervals for the edge-weights estimated in our models. The results clarify the precision in the estimation of the edge weights in the models.

PANELS C and D: Finally, we used the bootnet function to generate 1000 case-dropping bootstrapped samples, thereby allowing us to investigate the stability of the centrality estimates from our network models. The case-dropping bootstrapping results further suggested that even dropping the boot-strapped sample size down to 70% of the current sample continued to yield centrality estimates that correlated above .75 with those from the full sample in over 95% of the boot-strapped samples.

Supplemental Online Figure S3. *Centrality Coefficients from Each Model*

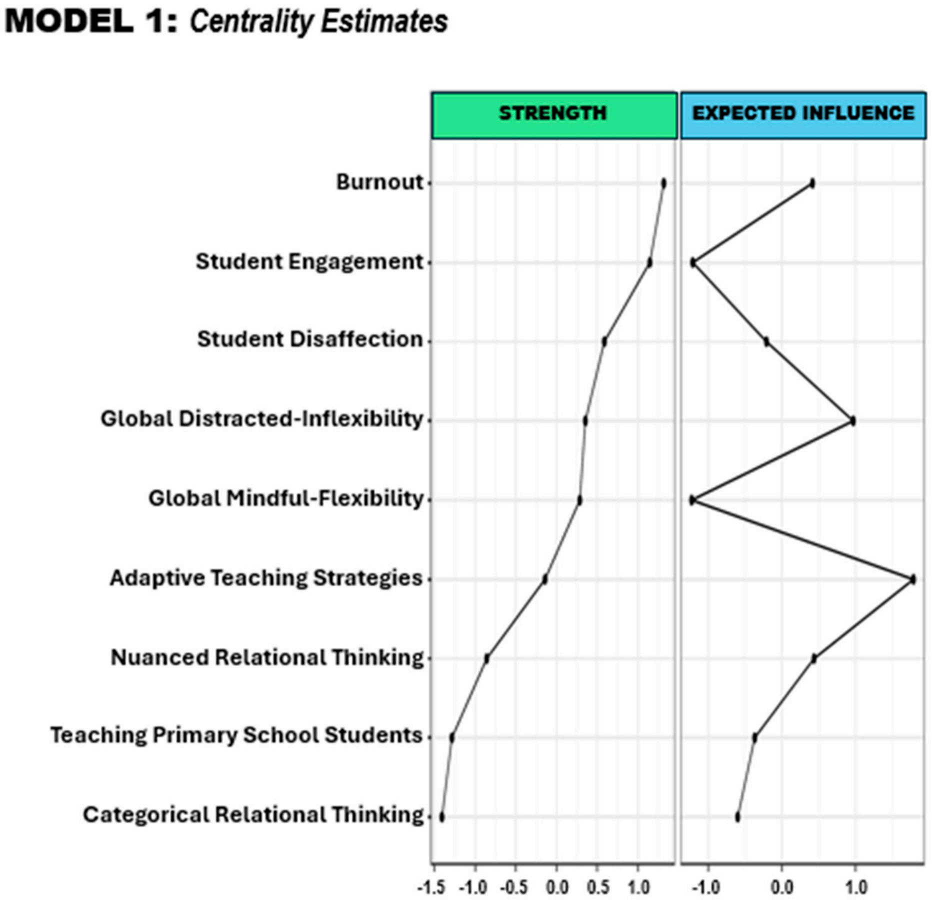

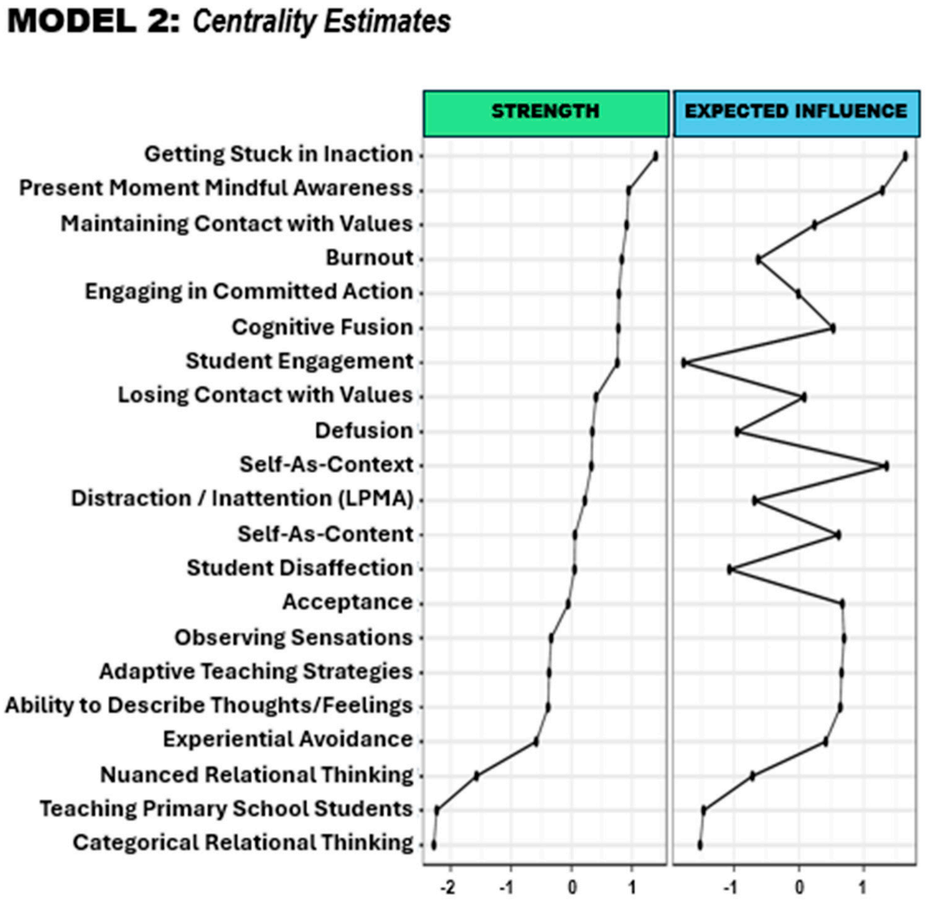

Supplement: Supplementary file 1 [file behavsci-16-01018-s001.zip › behavsci-4288902-supplementary.pdf]
